# Supplementary material for: Escherichia coli Strains Isolated from American Bison (Bison bison) Showed Uncommon Virulent Gene Patterns and Antimicrobial Multi-Resistance
Source: Microorganisms. 2024 Jul 3;12(7):1367. doi: 10.3390/microorganisms12071367 (PMC11278953; doi:10.3390/microorganisms12071367)
Supplement: Supplementary file 1 [file microorganisms-12-01367-s001.zip › microorganisms-3072143-supplementary.pdf]

**Supplementary Table S1.** Primer sequences and sizes of PCR products			
Gene	Sequence	Size	Reference
eae	3′-GGGGACATTTTAGTCA-5′/<br>5′-GATCAGAAGCGAGTTGTGG-3′	600	This study
***esc*V**	3′-GGCTCTCTTCTTTATGGCTG-5′/<br>5′-CCTTTTACAAACTTCATCGCC-3′	534	Karakaya et. al., 2022
***bfp*B**	3′-GATAAAACTGATACTGGGCAGC-5′/<br>5′-AGTGACTGTTCGGGAAGCAC-3′	826	Karakaya et. al., 2022
***stx*1**	3′-GGCGTTCTTATGTAATGACTGC-5′/<br>5′-ATCCCACGGACTCTTCCATC-3′	250	Karakaya et. al., 2022
***stx*2**	3′-CGTTTTGACCATCTTCGTCTG-5′/<br>5′-AGCGTAAGGCTTCTGCTGTG-3′	325	Karakaya et. al., 2022
st	3′-TAGAGACCGGTATTACAGAAATCTGA-5′/<br>5′-TCATCCCGAATTCTGTTATATATGTC-3′	282	This study
lt	3′-GGTTGGCAATTTTTATTTCTGTA-5′/<br>5′-ATTACAACAAAGTTCACAGCAGTA-3′	183	This study
aggr	3′-CTAATTGTACAATCGATGTGA-5′/<br>5′-ATGAAGTAATTCTTGAAT-3′	308	This study
ial	3′-GGTATGATGATGATGAGTCCA-5′/<br>5′-GGAGGCCAACAATTATTTCC-3′	650	This study
***chu*A**	5′-TGCCGCCAGTACCAAAGACA-3′/<br>5′-ATGGTACCGGACGAACCAAC-3′	288	Clermont et. al., 2013
***yja*A**	5′-CAAACGTGAAGTGTCAGGAG-3′/<br>5′-AATGCGTTCCTCAACCTGTG-3′	211	Clermont et. al., 2013
**TspE4c2**	5′-CACTATTCGTAAGGTCATCC-3′/<br>5′-AGTTTATCGCTGCGGGTCGC-3′	152	Clermont et. al., 2013
***arp*A1**	5′-AACGCTATTCGCCAGCTTGC-3′/<br>5′-TCTCCCCATACCGTACGCTA-3′	400	Clermont et. al., 2013
***arp*AgpE**	5′-GATTCCATCTTGTCAAAATATGCC-3′/<br>5′-GAAAAGAAAAAGAATTCCCAAGAG-3′	301	Clermont et. al., 2013

***trp*AgpC**	5′-AGTTTTATGCCCAGTGCGAG-3′/<br>5′-TCTGCGCCGGTCACGCCC-3′	219	Clermont et. al., 2013
***trp*BA**	5′-CGGCGATAAAGACATCTTCAC-3′/<br>5′-GCAACGCGGCCTGGCGGAAG-3′	489	Clermont et. al., 2013
